# Catheter Ablation for Brugada Syndrome

**DOI:** 10.19102/icrm.2021.120502

**Published:** 2021-05-15

**Authors:** Dingxin Qin, Weeranun D. Bode, E. Kevin Heist, Steven A. Lubitz, Pasquale Santangeli, Jeremy Ruskin, Moussa Mansour

**Affiliations:** ^1^Cardiac Arrhythmia Unit, Heart Center, Massachusetts General Hospital, Boston, MA, USA; ^2^Division of Cardiology, Electrophysiology Section, Hospital of the University of Pennsylvania, Philadelphia, PA, USA

**Keywords:** Brugada syndrome, catheter ablation, ventricular fibrillation

## Abstract

We report a case of catheter ablation of Brugada syndrome in a patient with refractory ventricular fibrillation despite quinidine therapy. We performed epicardial substrate mapping, which identified an area of abnormal fractionated, prolonged electrogram in the anterior right ventricular outflow tract. Warm saline infusion into the pericardial space induced further delay of the local electrogram, consistent with Brugada syndrome physiology. Coronary angiography confirmed that the area was distant from major coronary arteries. Ablation was performed in this area, which eliminated local abnormal electrograms and led to the disappearance of coved-type ST elevation in V1–V2. No ventricular fibrillation had recurred by five months of follow-up.

## Introduction

Previous studies have shown that Brugada syndrome (BrS) is associated with interstitial fibrosis and reduced gap-junction expression in the epicardial right ventricular outflow tract (RVOT).^1^ There is emerging evidence that epicardial substrate modification in the RVOT by catheter ablation could be an effective treatment for symptomatic drug-refractory BrS.^2^ However, the data regarding this topic are limited.

This report discusses the performance of catheter ablation for BrS in a patient with recurrent polymorphic ventricular tachycardia and ventricular fibrillation (PMVT/VF) despite pharmacological therapy.

## Case presentation

A 55-year-old woman with a history of BrS and subcutaneous implantable cardioverter-defibrillator (ICD) placement on quinidine was admitted for recurrent episodes of out-of-hospital PMVT/VF and ICD shocks. Device interrogation showed three sequential episodes of PMVT/VF within 15 minutes that triggered six ICD shocks in total, including one episode that persisted through four ICD shocks before its spontaneous termination. In light of the severity of these events despite the patient being on quinidine therapy, she was scheduled for catheter ablation. Quinidine was held for five days prior to the procedure.

**Figure 1:**
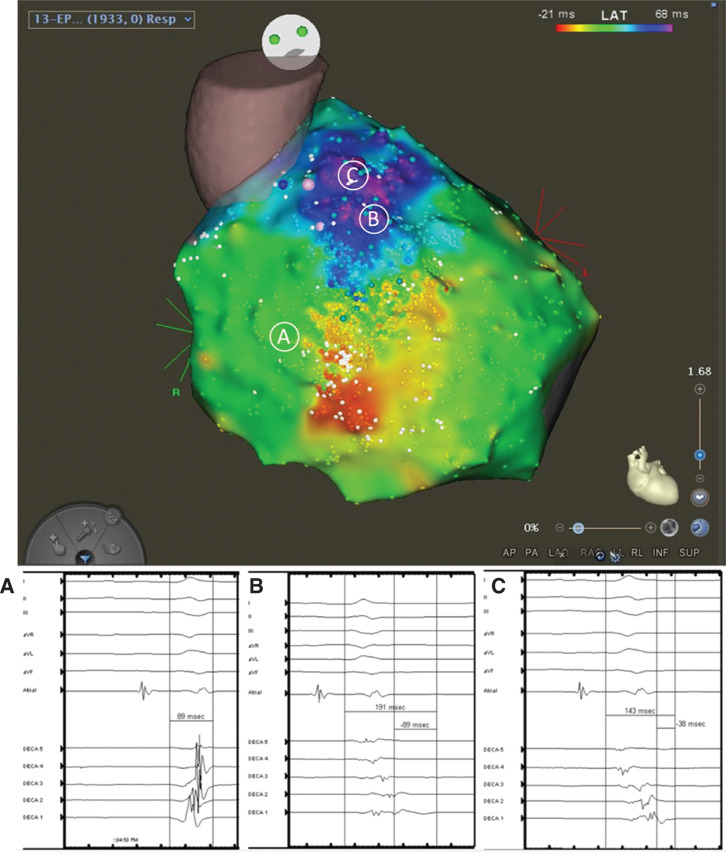
Epicardial activation mapping of the right ventricle. **A:** Normal RV electrogram duration was measured from the beginning of the QRS complex. **B and C:** Abnormal electrogram duration in the RVOT area, which extended beyond the end of the QRS complex, was measured from both the beginning and the end of the QRS complex. The A, B, and C in circles above correspond to the three electrograms below from left to right.

**Figure 2:**
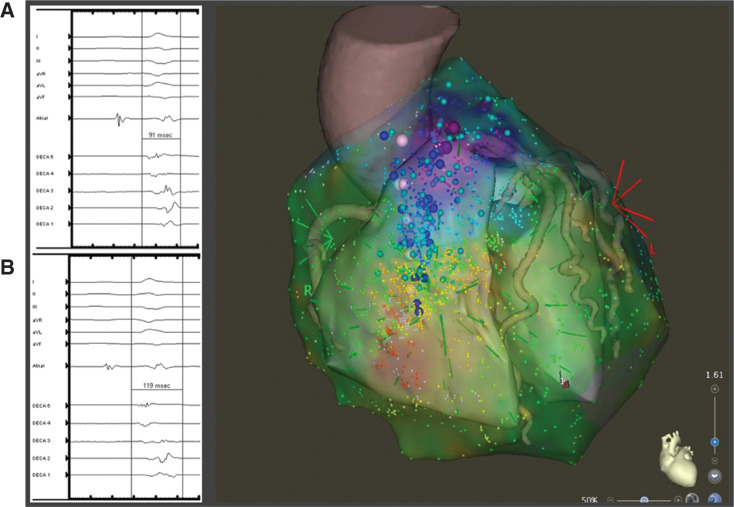
Integration of the epicardial activation map with a CARTOSOUND^®^ image and 3D CT scan with RVOT electrograms before **(A)** and after **(B)** pericardial warm saline infusion. The electrogram became more delayed and fractionated after warm saline infusion. The duration of the electrogram was measured from the beginning of the QRS complex.

**Figure 3:**
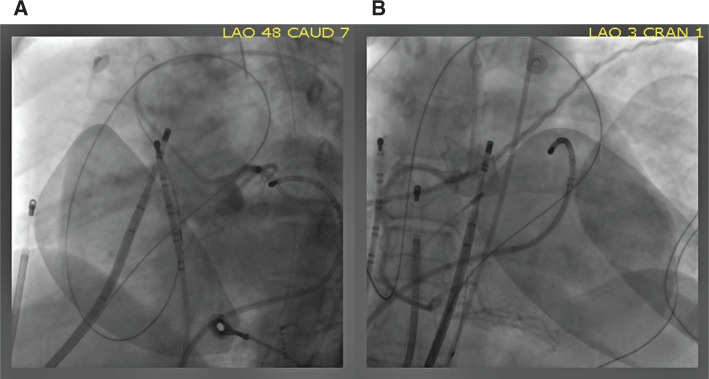
Left and right coronary angiograms before ablation. The ablation catheter was placed in the epicardial RVOT area at a safe distance from the left **(A)** and right **(B)** coronary arteries.

**Figure 4:**
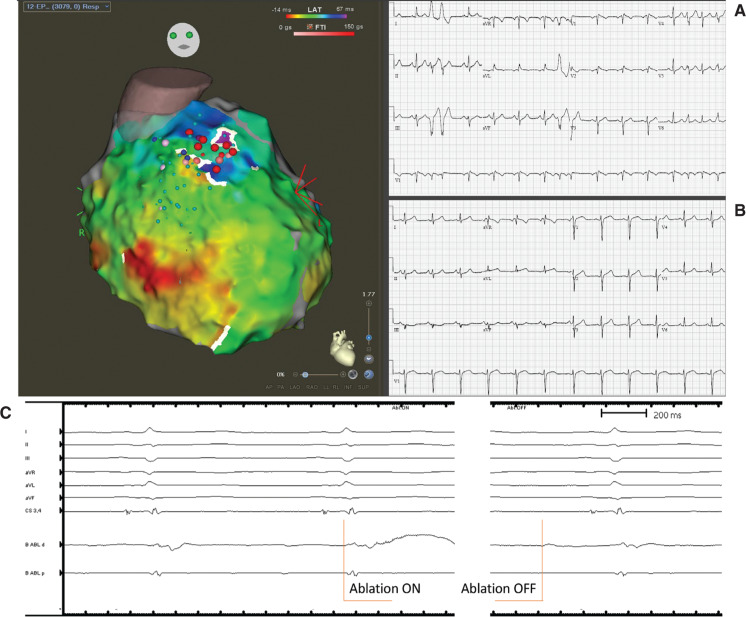
Epicardial activation map postablation. Ablation lesions are shown. The fractionated delayed electrograms in the RVOT area were eliminated. As compared with the preablation ECG **(A)**, the postablation ECG **(B)** showed the disappearance of the coved-type ST elevation in V1–V2. **C:** An example of an electrogram on the ablation catheter before and after ablation.

The ablation procedure was performed under general anesthesia. The pericardial space was accessed anteriorly using a micro-puncture apparatus, which was subsequently exchanged with a deflectable sheath (Agilis; Abbott, Chicago, IL, USA). A linear multipolar mapping catheter (DECANAV^®^; Biosense Webster, Diamond Bar, CA, USA) was used to map the epicardial surface. Voltage and sinus activation maps **(Figure 1)** were obtained (CARTO^®^ 3; Biosense Webster). Areas of delayed, fractionated, and prolonged potentials were annotated on the epicardial substrate map and were concentrated over the RVOT. Intracardiac echo was also used to reconstruct a three-dimensional (3D) shell of the endocardium of both ventricles (CARTOSOUND^®^; Biosense Webster). The epicardial and endocardial maps were then integrated with a 3D computed tomography (CT) image of the aortic root and coronary arteries **(Figure 2)**. After the location and timing of the areas of delayed activation were identified, 60 mL of warm saline (102°F) was injected into the epicardial space. This resulted in further delay of the abnormal electrical activity in the RVOT that lasted approximately five minutes, consistent with BrS pathophysiology **(Figures 2A and 2B)**. Left and right coronary angiograms were recorded to ensure a safe distance existed between the targeted area in the epicardial RVOT and the proximal coronary arteries **(Figure 3)**. Ablation targeted the fractionated potentials with delayed activation timing beyond the end of the QRS complex **(Figure 4)**, using radiofrequency energy up of 40 W (STSF; Biosense Webster), which was titrated down for a significant decrease or increase in impedance. The duration of RF delivery was 30 seconds for each lesion, with the endpoint being the loss of late potentials. After ablation, epicardial voltage and activation maps were acquired and demonstrated the elimination of the late abnormal local electrical activity **(Figure 4)**. No premature ventricular contractions were seen during the procedure. Postprocedure electrocardiography (ECG) confirmed the disappearance of the coved-type ST elevation in V1–V2 **(Figure 4)**. The patient recovered well and experienced no further PMVT/VF episodes to five months of follow-up.

## Discussion

This case illustrates an example of successful epicardial RVOT substrate modification in a patient with BrS. It highlights the important fact that, at least in a subset of cases, the pathological substrate in this disease is localized to a relatively small area in the epicardial RVOT, the elimination of which could significantly reduce the risk of future VT/VF events.

Early studies on the pathogenesis of BrS focused on the role of ion channel mutations, especially in the *SCN5A* gene, which can lead to repolarization abnormalities in the RVOT area.^3^ Treatment with quinidine, an I_to_ blocker, was effective in preventing VT/VF events in a proportion of patients. Recent studies have demonstrated that BrS is associated with structural alterations and electrical remodeling in the epicardial RVOT area,^1,4,5^ which plays an essential role in the pathogenesis of PMVT/VF events. Attempts at modifying the abnormal substrate in the epicardial RVOT with catheter ablation have been previously reported **(Table 1)**.^2,6–10^ Other ablation strategies, including endocardial ablation or the ablation of VF-triggering premature ventricular complexes,^2^ in combination with epicardial ablation, have also have been described, with the success of the procedure seemingly realized mostly by the epicardial ablation. The abnormal intracardiac electrical activity in BrS can be subtle and probably dynamic. Revealing the epicardial substrate and provoking the BrS phenotype is an important part of the ablation procedure. This can be performed by the infusion of sodium channel blockers. In patients with a high VF burden, where the infusion of these medications can precipitate an arrhythmia storm, intrapericardial warm saline can be used and has a transient effect.

The endpoints of the procedure constitute another important consideration. The elimination of local delayed electrical activity is generally feasible because the targeted area tends to be relatively small and reasonably far from the coronary arteries in most patients. Another endpoint is the elimination of the BrS pattern on the surface 12-lead ECG. This is an important endpoint; the persistence of BrS after ablation has been associated with VT/VF recurrence, while its disappearance was predictive of good clinical outcomes.^2,6–9^ However, it is important to keep in mind that elimination of the BrS ECG pattern may not be obvious immediately after the procedure because the ECG can be affected by inflammation and pericarditis, which can present as ST-segment abnormalities, and the normalization may take weeks to become obvious. The inducibility of VT/VF postablation as an endpoint remains controversial.^2^ The Programmed Electrical Stimulation Predictive Value (PRELUDE) registry involving 308 patients with spontaneous or drug-induced BrS type I pattern and no prior history of cardiac arrest showed that VT/VF inducibility was unable to identify high-risk patients.^11^ A 2016 pooled analysis suggested that the induction of VT/VF with fewer extrastimuli was associated with a greater event risk, but clinical risk factors were more important determinants for arrhythmia risk.^12^

## Conclusion

Successful catheter ablation for BrS can be achieved by targeting the abnormal substrate in the epicardial anterior RVOT. Multicenter studies are needed to compare different mapping and ablation strategies, to define the endpoint of catheter ablation, and to confirm the effectiveness of this approach.

## Figures and Tables

**Table 1:** Case Series of Epicardial with and without Endocardial Mapping and Catheter Ablation for BrS.

Study	N	Mapping	Ablation Site	Provocation Agent	VT/VF Preablation	VT/VF Freedom Postablation	Adverse Events
Nademanee et al.^6^	9	Epi + Endo substrate	Epi RVOT	Ajmaline	9 (100%)	8 (89%)	Pericarditis (n = 2)
Brugada et al.^7^	14	Epi ± Endo substrate	Epi RV and RVOT	Flecainide	14 (100%)	14 (100%)	Pericarditis (n = 1)
Zhang et al.^8^	11	Epi + Endo substrate	Epi RVOT	Propafenone or procainamide	11 (100%)	8 (73%)	Pericarditis (n = 2)
Chung et al.^9^	15	Epi + Endo substrate + PVC	Epi RVOT and PVC	Epicardial warm water	15 (100%)	14 (93%)	None
Pappone et al.^10^	135	Epi + Endo substrate	Epi RV and RVOT	Ajmaline	135 (100%)	133 (98.5%)	Pericardial effusion (n = 5)

BrS: Brugada syndrome; Endo: endocardial; Epi: epicardial; RVOT: right ventricular outflow tract; VF: ventricular fibrillation; VT: ventricular tachycardia.
